# The emergence of common health conditions across the life course: evidence from the Born in Bradford family cohort

**DOI:** 10.12688/wellcomeopenres.20992.2

**Published:** 2025-05-16

**Authors:** Gillian Santorelli, Dan Lewer, Krishnarajah Nirantharakumar, Siang Ing Lee, Katherine Phillips, Rosemary R.C. McEachan, John Wright

**Affiliations:** 1 Bradford Institute for Health Research, Bradford Teaching Hospitals NHS Foundation Trust, Bradford, England, BD9 6RJ, UK; 2 Institute for Applied Health Research, University of Birmingham, Birmingham, England, UK

**Keywords:** Born in Bradford, health conditions, ethnicity, prevalence, incidence, hazard ratios, common mental disorders, diabetes

## Abstract

**Background:**

Born in Bradford (BiB) is a family cohort study with linked routine health records. We calculated the rates of common health conditions and explored differences between White European and South Asian participants.

**Methods:**

21 health conditions were identified using diagnostic codes and prescription records extracted from electronic health records. We calculated 2-year period prevalence before recruitment and incidence rates per 1000 person-years were calculated from recruitment to the end of 2021 (or censoring). Age-adjusted Cox proportional hazard models estimated hazard ratios (HR) by ethnicity.

**Results:**

The sample included 9,784 mothers, 52% were of South Asian heritage and 48% were White European. The highest prevalence and incidence rates were observed for common mental health disorders and eczema. South Asian women had higher incidence of 14 conditions, including diabetes (HR 3.94 [95% CI 3.15, 4.94]), chronic liver disease (2.98 [2.29, 3.88]) and thyroid disorders (1.87 [1.50, 2.33]), but lower incidence of cancer (0.51 [0.38, 0.68]), other and common mental health disorders (0.56 [0.45, 0.71] and 0.69 [0.64, 0.74] respectively), and other neuromuscular conditions (0.63 [0.49, 0.82]).

**Conclusions/discussion:**

This study reveals significant differences in the occurrence of several non-communicable health conditions between White European and South Asian women. The observed higher incidence of several conditions in South Asian women, consistent with established knowledge regarding elevated risks for diseases such as diabetes, likely reflects the complex interplay of social, cultural, lifestyle, environmental, and genetic determinants. These findings emphasise the need for culturally sensitive and targeted public health interventions aimed at addressing modifiable risk factors at both the individual and systemic levels to alleviate the burden of long-term health conditions and reduce existing health inequalities.

## Plain language summary

Born in Bradford is a research study which aims to find out what keeps families healthy by tracking the lives of over 40,000 people living in Bradford, which is a city in the north of England. We looked at the medical history of nearly 10,000 mothers taking part in the study to see how many had developed one or more of 21 health conditions since they joined the study. We found that women of South Asian heritage were more likely to develop diabetes, chronic liver disease, and thyroid disorders compared to white women, but were less likely to develop cancer, common mental health disorders and neuromuscular conditions such as fibromyalgia. These differences may be explained by social, cultural, lifestyle, environmental and genetic factors, and it is important to understand why this happens. By doing so, we can try to address potential risk factors for these diseases to reduce health inequalities and prevent people from living with long-term health conditions.

## Introduction

Poverty and deprivation severely impact women's health in the UK, reducing life expectancy and diminishing the number of years lived in good health, particularly within disadvantaged communities ^1^. These health disparities are often exacerbated for women from Black, Asian, and other minority ethnic backgrounds, who frequently contend with intersecting inequalities tied to both socioeconomic status and ethnicity ^2^. Factors such as poor living conditions, chronic stress, limited access to healthcare and adverse environmental exposures accumulate across the lifespan ^3^, contributing to long-term physical and mental health problems, including those arising during the perinatal period, while hindering opportunities for healthy living. Addressing both poverty and systemic ethnic inequalities is therefore essential for improving women's overall and reproductive health.

Bradford, a city in West Yorkshire, England, faces significant challenges related to these issues. Characterised by a young population, high levels of deprivation (ranking 13 ^th^ most deprived local authority out of 317 in England, with almost a fifth of children living in low income families) ^4^, and notable ethnic diversity, with 57% identifying as White British and 26% of Pakistani heritage ^5^. Concerns regarding high rates of illness in Bradford led to the establishment of the Born in Bradford (BiB) cohort study. Between 2007 and 2010, the BiB study recruited 12,453 women during 13,776 pregnancies (resulting in 13,858 births) to investigate how socioeconomic, environmental, behavioural, and genetic factors influence health and well-being ^6^. As these mothers now enter middle age, the BiB cohort presents a unique opportunity to examine the accumulation of health conditions following childbirth. This study therefore aimed to (1) describe the rates of common non-communicable health conditions among mothers in the BiB cohort; and (2) determine if the incidence of these conditions differs between the two largest ethnic groups in Bradford.

## Methods

### Participants and data sources

This study utilised data from the BiB cohort. Between 2007 and 2010, BiB enrolled 12,453 pregnant women during 13,776 pregnancies who were booked for delivery at the Bradford Royal Infirmary, resulting in 13,858 births ^6^. At recruitment, participants completed a baseline questionnaire, providing demographic information, and consented to the linkage of their electronic health records (EHR). Linkage to routine health data has been successfully achieved for over 98% of the cohort participants. For this study, primary care data containing information on health conditions were extracted from SystmOne, the clinical EHR system used universally across all GP practices in Bradford. This study also leverages a collaboration between BiB and MuM-PreDiCT, a UK-wide research project focused on pregnant women with two or more long-term health conditions ^7^.

### Outcomes

We adopted a list of 79 long-term health conditions identified by MuM-PreDiCT as important factors in multimorbidity, originally selected for their prevalence, potential to impact on pregnancy outcomes, patient importance, and data availability. For the present study, while our exploratory analysis assessed the incidence of all 79 health conditions within the BiB cohort, we report trends for 21 conditions (19 physical and 2 mental health) with an incidence of ≥2% per 000 person-years in at least one of the major ethnic groups in Bradford. Many of these 21 health conditions aggregate several diagnoses; see Table 1 for details and definitions.

**Table 1.** Health conditions examined in BiB participants, with phenome definition.

Health condition	Conditions included (where applicable)	Definition
Cancer	- All cancers	Diagnosis code
Hypertension	N/A	Diagnosis code
Eczema	N/A	Diagnosis code OR ≥ 2 related prescriptions (BNF section 13.4)
Other skin disorders	- Seborrheic dermatitis - Rosacea - Hidradenitis suppurativa - Lichen planus	Diagnosis code
Allergic rhinoconjunctivitis	- Allergic rhinitis (hay fever) - Allergic conjunctivitis	Diagnosis code OR ≥ 1 related prescription during hay fever season (1 ^st^ March to 31 ^st^ July) of anti- histamines or intra-nasal corticosteroids (BNF section 12.2.1)
Irritable bowel syndrome	N/A	Diagnosis code
Chronic liver disease	- Chronic hepatitis B & C - Alcoholic liver disease - Autoimmune liver disease - Cirrhosis - Non-alcoholic fatty liver disease	Diagnosis code
Gynaecological disorders	- Polycystic ovarian syndrome - Endometriosis - Uterine fibroids - Infertility	Diagnosis code
Pelvic floor dysfunction	- Urinary incontinence - Faecal incontinence - Genital prolapse	Diagnosis code
Rheumatological conditions	- Systemic lupus erythematosus - Spondyloarthritis (psoriatic arthritis, ankylosing spondylitis) - Inflammatory arthritis (rheumatoid arthritis, Sjögren's syndrome, Raynaud’s syndrome, systemic sclerosis, primary systemic vasculitis) - Ehler’s Danlos Syndrome type 3 (hypermobile)	Diagnosis code
Orthopaedic conditions	- Scoliosis - Vertebral disorder (intervertebral disc disorder, spondylosis, spondylolisthesis, collapsed vertebrae, spinal stenosis) - Chronic back pain - Osteoporosis - Osteoarthritis	Diagnosis code
Migraine	N/A	Diagnosis code
Other chronic headaches	- Tension-type headaches - Cluster headaches	Diagnosis code
Peripheral neuropathy	N/A	Diagnosis code
Asthma	N/A	Diagnosis code OR ≥3 prescriptions of bronchodilators or inhaled corticosteroids (BNF section 3.2.1 and 3.1.1)
Gallstones	N/A	Diagnosis code
Diabetes	- Type 1 diabetes - Type 2 diabetes	Diagnosis code OR ≥4 antidiabetic prescriptions within each calendar year (BNF sections 6.1 and 6.1.2)
Thyroid disorders	- Hyperthyroidism - Hypothyroidism	Diagnosis code
Other neuromuscular disorders	- Chronic fatigue syndrome/ myalgic encephalomyelitis - Fibromyalgia - Chronic pain syndrome	Diagnosis code
Common mental health disorder (CMHD)	- Depression - Anxiety (including phobia, panic disorder, and post-traumatic stress disorder) - Antidepressant or anxiolytic prescriptions without a diagnosis of depression or anxiety	Diagnosis code or ≥4 anti-depressant or anxiolytic prescriptions (BNF sections 4.1.2, 4.3 [excluding amitriptyline], Propranolol 10mg or 40mg)
Other mental illness	- Obsessive compulsive disorder - Personality disorder - Dissociative disorder - Self-harm (including suicide)	Diagnosis code

### Ethnicity

Information on ethnicity was obtained from research questionnaires and routine data sources, all of which were self-reported by the participant and categorised using the same classification as the 2001 UK census ^8^. White European ethnicity included those who identified as White British, Irish, Gypsy/Irish traveller, and White Other; South Asian ethnicity was assigned to participants of Pakistani, Indian, and Bangladeshi heritage.

### Ethics statement

Ethics approval for the Born in Bradford study was granted by the National Health Service Health Research Authority Yorkshire and the Humber (Bradford Leeds) Research Ethics Committee (reference: 07/H1302/112, date of approval 01/04/2008). Informed consent for data collection and linkage to routine healthcare records was provided by participants at recruitment to the BiB cohort study. Further information on our privacy policy can be found here: https://borninbradford.nhs.uk/privacy-policy/.

### Statistical analysis

To ensure sufficient data for identifying existing health conditions, we excluded participants with fewer than two years of medical records prior to recruitment. Baseline prevalence for each condition was defined as having either a GP-recorded diagnostic code or a relevant prescription during the two years before the recruitment date (“time 0”). Prevalence was calculated separately for each ethnic group and is reported as percentages. Incidence rates per 1,000 person years (IR/1000) were calculated from the date of recruitment, with follow-up ending at the earliest of: last GP-recorded event (up to 31 ^st^ December 2021), withdrawal from the study, relocation out of the area, or death. Individuals with prevalent cases were excluded from the incidence calculations. For the main categories of conditions, we used age-adjusted Cox proportional hazards models to estimate hazard ratios (HR), comparing the risk of developing each health condition between South Asian and White European participants (the reference group). An HR of 1 indicates no difference in risk between groups; values above 1 suggests a higher risk among South Asians, while values below 1 suggest a lower risk. HR were not calculated for individual conditions within broader categories due to small sample sizes. Analyses were conducted using Stata/SE version 17 software ( https://www.stata.com/).

## Results

**Figure 1.**
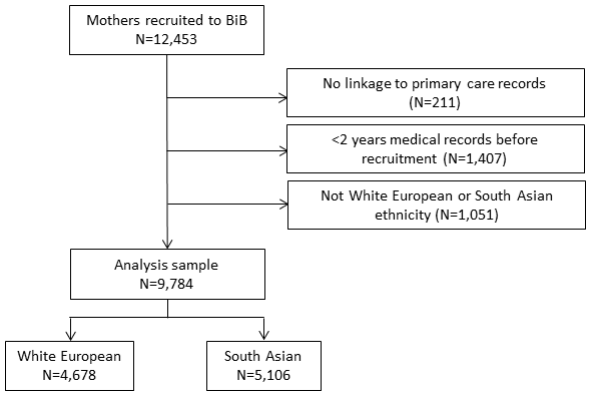
Sample flow chart.

The sample comprised 9,784 mothers, of whom 52% were of South Asian ethnicity; see Figure 1 for further details of the sample selection, and Table 2 for participant characteristics.

**Table 2.** Participant characteristics.

Characteristic	All (n=9,784)	White European (n=4,678)	South Asian (n=5,106)
Year of recruitment, n (%)			
2007	2,286 (23.4)	1,076 (23.0)	1,210 (23.7)
2008	2,740 (28.0)	1,276 (27.3)	1,464 (28.7)
2009	2,617 (26.7)	1,239 (26.5)	1,378 (27.0)
2010	2,141 (21.9)	1,087 (23.2)	1,054 (20.6)
Age at recruitment, mean (SD)	27.7 (5.7)	26.8 (6.1)	28.5 (5.1)
BMI, kg/m ^2^, mean (SD)	26.2 (5.7)	26.6 (6.0)	25.8 (5.5)
Missing, n (%)	1,791 (18.3)	807 (17.3)	984 (19.3)
Smoked during pregnancy, n (%)			
No	7,033 (71.9)	2,787 (59.6)	4,246 (83.2)
Yes	1,573 (16.1)	1,408 (30.1)	165 (3.2)
Missing	1,178 (12.0)	483 (10.3)	695 (13.6)
IMD, n (%)			
1 (most deprived)	6,364 (65.0)	2,452 (52.4)	3,912 (76.6)
2	1,775 (18.1)	1,004 (21.5)	771 (15.1)
3	1,120 (11.4)	782 (16.7)	338 (6.6)
4/5 (least deprived)	466 (4.8)	416 (8.9)	50 (1.0)
Missing	59 (0.6)	24 (0.5)	35 (0.7)

The prevalence and IR/1000 for each condition are presented in Table 3. The most prevalent conditions among White European and South Asian women were common mental health disorders (29.3% vs 15.7% respectively), eczema (25.3% vs 26.4%), and asthma (22.5% vs 9.1%). The highest IR/1000 was observed for common mental health disorders (55.9 vs 35.4 in White European and South Asian women respectively). High IR/1000 was observed in South Asian women for eczema, allergic rhinoconjunctivitis, and migraine. For the individual health conditions grouped within broader categories, the IR/1000 were statistically significantly higher in White European women compared to South Asian women for fibromyalgia, endometriosis, genital prolapse, and all conditions classified under common and other mental health disorders apart from dissociative disorders. Conversely, South Asian women had higher rates of hypothyroidism, seborrhoeic dermatitis, lichen planus, systemic lupus erythematosus, back pain, osteoarthritis, polycystic ovarian syndrome, uterine fibroids, infertility, urinary incontinence, and all conditions included in other chronic headaches.

**Table 3.** Prevalent cases (%) and incidence rates per 1000 person years (IR/1000) for broader health conditions and the conditions included within them, stratified by ethnic group.

Health condition	Prevalence %		IR/1000 ^b^	
	White European (n=4,678)	South Asian (n=5,106)	White European (n=4,678)	South Asian (n=5,106)
**Common mental health disorders**	**29.3**	**15.7**	**55.9**	**35.4**
* Depression*	*22.5*	*10.9*	*39.5*	*25.1*
* Anxiety*	*14.3*	*8.8*	*23.6*	*15.3*
* Antidepressants/anxiolytics* *drugs only ^a^*	*0.6*	*0.2*	*2.9*	*1.3*
**Migraine**	**11.1**	**10.4**	**10.1**	**13.1**
**Allergic rhinoconjunctivitis**	**19.7**	**18.3**	**14.9**	**23.6**
**Eczema**	**25.3**	**26.4**	**23.9**	**25.3**
**Asthma**	**22.5**	**9.1**	**5.9**	**6.9**
**Other chronic headaches**	**4.6**	**6.0**	**5.9**	**8.8**
* Cluster headaches*	*0.3*	*0.1*	*0.1*	*0.2*
* Tension headaches*	*4.3*	*5.9*	*5.7*	*8.5*
* Other headaches*	*0*	*0.2*	*0.3*	*0.6*
**Pelvic floor dysfunction**	**1.9**	**2.3**	**6.7**	**8.7**
* Urinary incontinence*	*1.5*	*1.8*	*5.2*	*7.2*
* Faecal incontinence*	*0.4*	*0.1*	*0.2*	*0.3*
* Genital prolapse*	*0.2*	*0.5*	*2.4*	*2.0*
**Diabetes**	**0.5**	**0.6**	**1.7**	**7.5**
**Peripheral neuropathy**	**1.2**	**2.1**	**4.6**	**6.4**
**Orthopaedic conditions**	**1.4**	**1.8**	**5.0**	**6.6**
* Vertebral disorder*	*0.8*	*0.5*	*2.0*	*2.3*
* Scoliosis*	*0.2*	*0.1*	*0.0*	*0.0*
* Back pain*	*0.4*	*0.9*	*1.3*	*2.0*
* Osteoporosis*	*0.0*	*0.1*	*0.2*	*0.2*
* Osteoarthritis*	*0.2*	*0.4*	*2.0*	*3.1*
**Thyroid disorder**	**1.7**	**2.3**	**2.3**	**4.4**
* Hyperthyroidism*	*0.5*	*0.7*	*0.7*	*0.9*
* Hypothyroidism*	*1.4*	*1.8*	*1.9*	*3.6*
**Gynaecological conditions**	**9.5**	**14.9**	**4.8**	**7.8**
* Polycystic ovaries syndrome*	*2.0*	*3.0*	*1.1*	*1.5*
* Endometriosis*	*1.4*	*0.6*	*1.3*	*0.8*
* Fibroids*	*0.4*	*0.7*	*1.7*	*4.3*
* Infertility*	*7.2*	*13.0*	*1.5*	*2.0*
**Hypertension**	**1.4**	**1.3**	**3.9**	**6.3**
**Other skin disorders**	**3.9**	**5.1**	**4.3**	**5.5**
* Rosacea*	*0.7*	*0.4*	*1.2*	*1.1*
* Seborrheic dermatitis*	*2.7*	*4.3*	*2.3*	*3.4*
* Hidradenitis*	*0.5*	*0.4*	*0.7*	*0.6*
* Lichen planus*	*0.1*	*0.2*	*0.2*	*0.6*
**Neuromuscular conditions**	**0.5**	**0.4**	**2.6**	**1.8**
* Chronic fatigue syndrome*	*0.2*	*0.1*	*0.1*	*0.0*
* Fibromyalgia*	*0.2*	*0.3*	*2.1*	*1.3*
* Complex pain syndrome*	*0.2*	*0.1*	*0.6*	*0.5*
**Other mental health conditions**	**9.9**	**4.5**	**4.2**	**2.1**
* Obsessive compulsive disorder*	*0.5*	*0.1*	*0.6*	*0.3*
* Personality disorder*	*0.5*	*0.0*	*0.7*	*0.2*
* Dissociative disorder*	*0.1*	*0.1*	*0.3*	*0.3*
* Self-harm*	*9.2*	*4.3*	*3.3*	*1.5*
**Irritable bowel syndrome**	**6.2**	**3.4**	**4.2**	**3.4**
**Rheumatological conditions**	**1.5**	**1.0**	**2.2**	**2.3**
* Systemic lupus erythematosus*	*0.0*	*0.0*	*0.1*	*0.2*
* Spondyloarthritis*	*0.0*	*0.1*	*0.6*	*0.3*
* Inflammatory arthritis*	*1.2*	*0.8*	*1.4*	*1.7*
* Ehler's-Danlos syndrome*	*0.3*	*0.1*	*0.3*	*0.2*
**Gallstones**	**1.0**	**1.1**	**2.7**	**3.5**
**Chronic liver disease**	**0.3**	**1.1**	**1.3**	**4.4**
* Hepatitis B & C*	*0.2*	*0.9*	*0.2*	*0.2*
* Fatty liver*	*0.1*	*0.2*	*0.9*	*4.1*
* Other liver disease*	*0.2*	*0.9*	*0.4*	*0.3*
**Cancer**	**0.7**	**0.5**	**2.3**	**1.3**

*^a^ Prescribed in the absence a diagnosis of depression or anxiety. ^b^ Please note that the IR/1000 for a broader health condition may not equal the total of the conditions included within them due to some individuals having more than one of the individual conditions, or because of rounding*.

Cumulative hazards plots for the health condition examined are presented in Figure 2a to Figure 2e and show the different patterns of the conditions over time by ethnic group. Some had very similar trajectories over the whole study period (rheumatological and orthopaedic conditions, cancer, irritable bowel syndrome and asthma); some showed ethnic divergence from the birth of the BiB child (eczema, allergic rhinoconjunctivitis); some conditions diverged in the early years (other skin, thyroid and gynaecological disorders, migraine/other headaches, diabetes); whilst others diverged between 7 – 10 years after recruitment (gallstones, hypertension, peripheral neuropathy, pelvis floor dysfunction and neuromuscular disorders). The increasing divergence in the rate of common mental health disorders in WE compared to SA women was apparent shortly after recruitment, whereas other mental health disorders had a similar trajectory in both ethnic groups until 2 – 3 years after recruitment before increasing more rapidly in WE women.

**Figure 2a.**
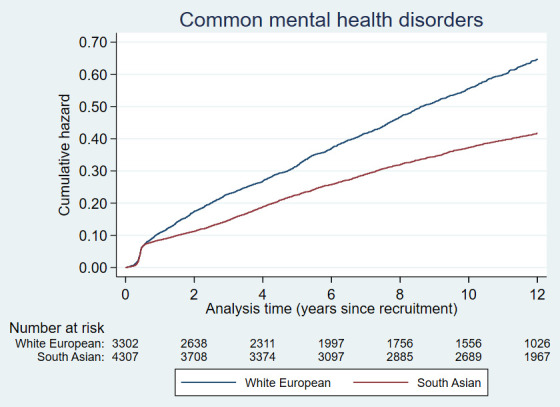
Health conditions with a cumulative hazard >30%.

**Figure 2b.**
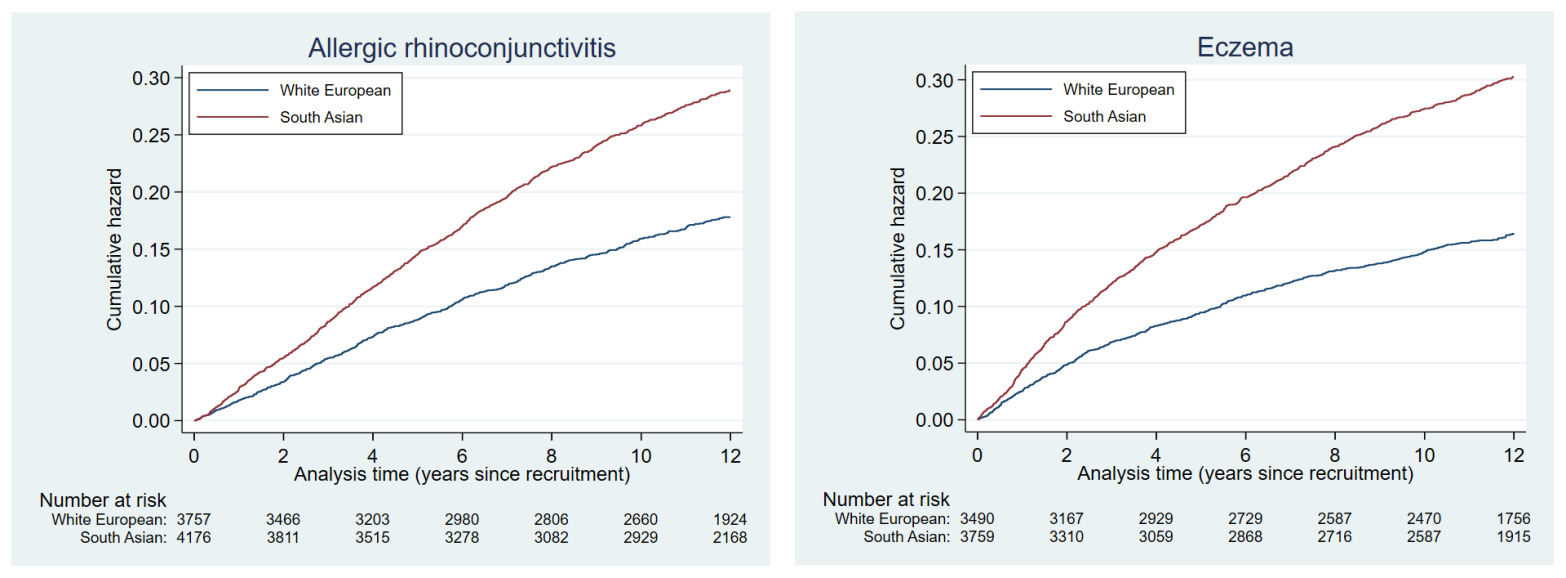
Health conditions with a cumulative hazard of 21% to 30%.

**Figure 2c.**
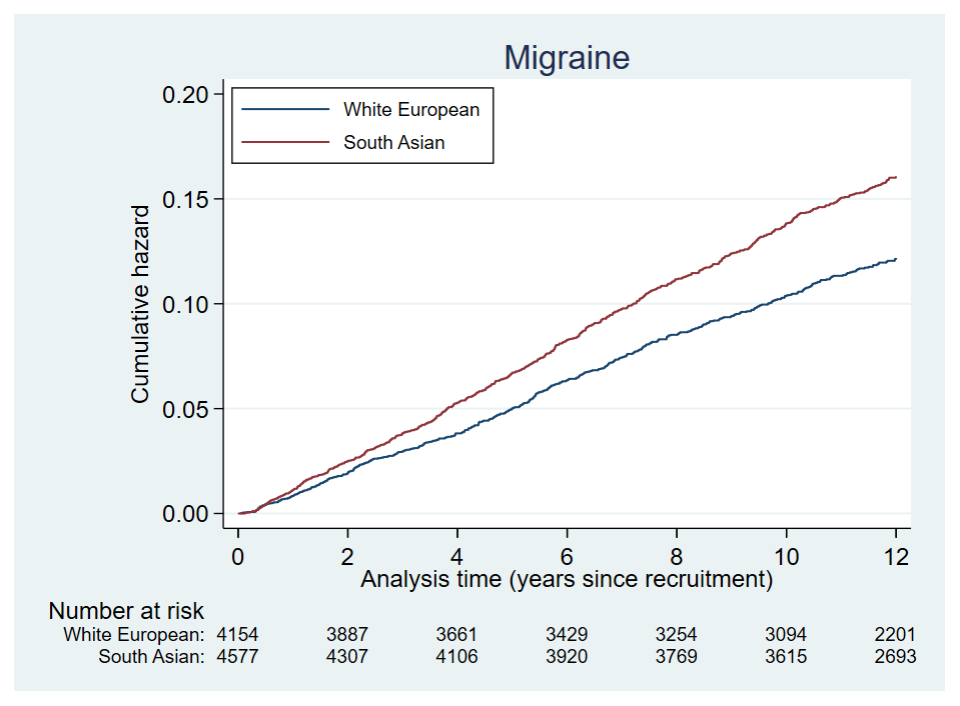
Health conditions with a cumulative hazard of 11% to 20%.

**Figure 2d.**
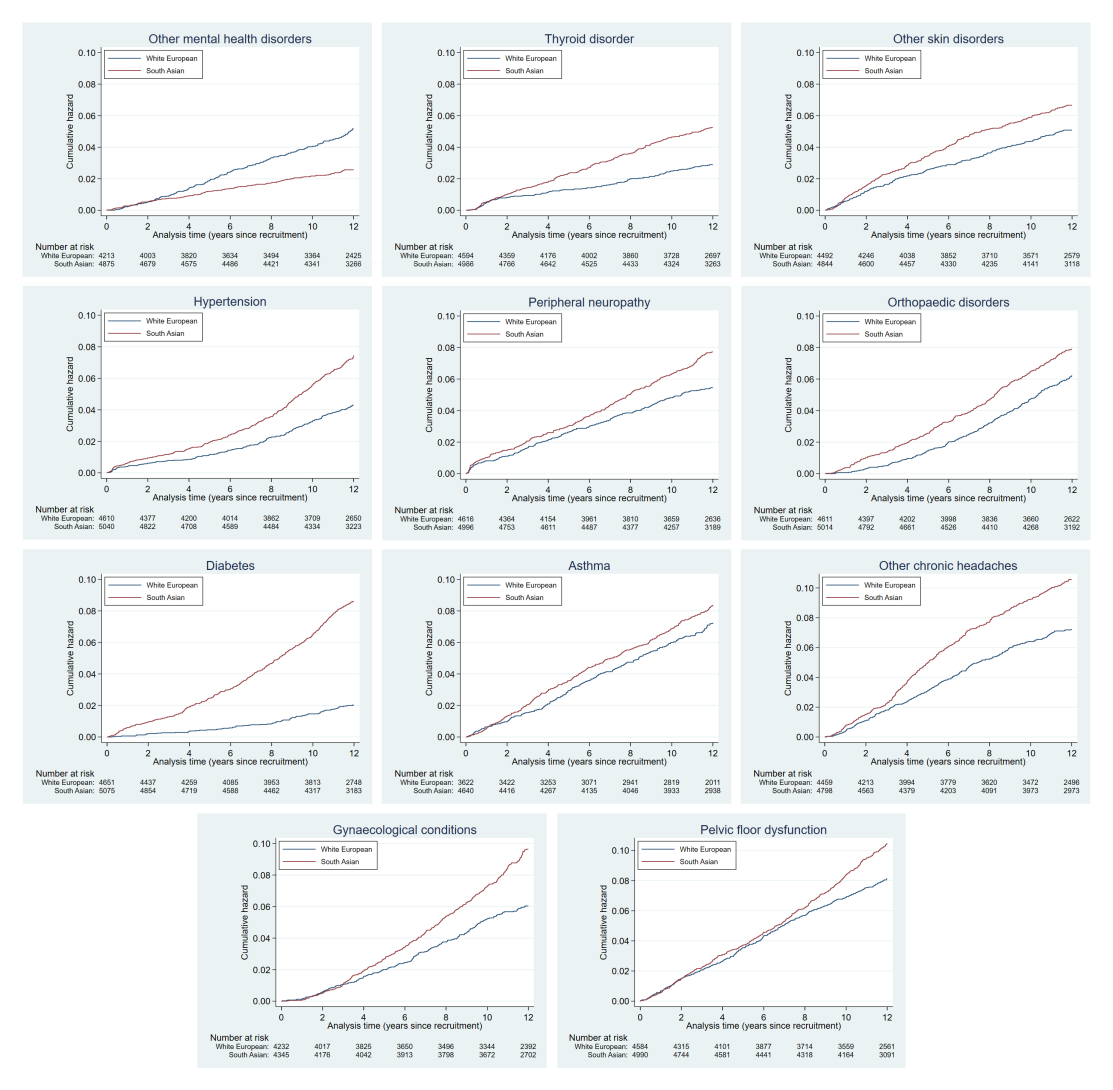
Health conditions with a cumulative hazard of 6% to 10%.

**Figure 2e.**
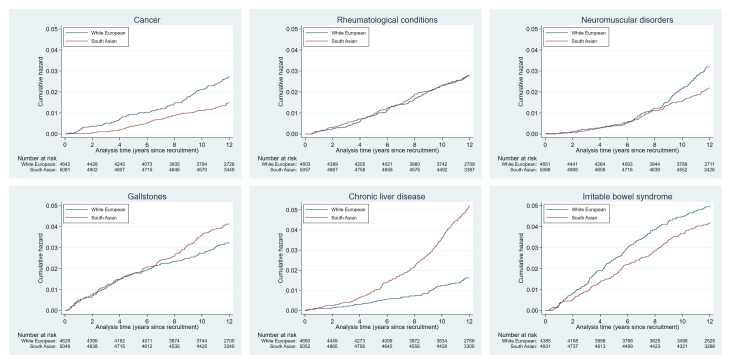
Health conditions with a cumulative hazard 2% to 5%.

**Figure 3.**
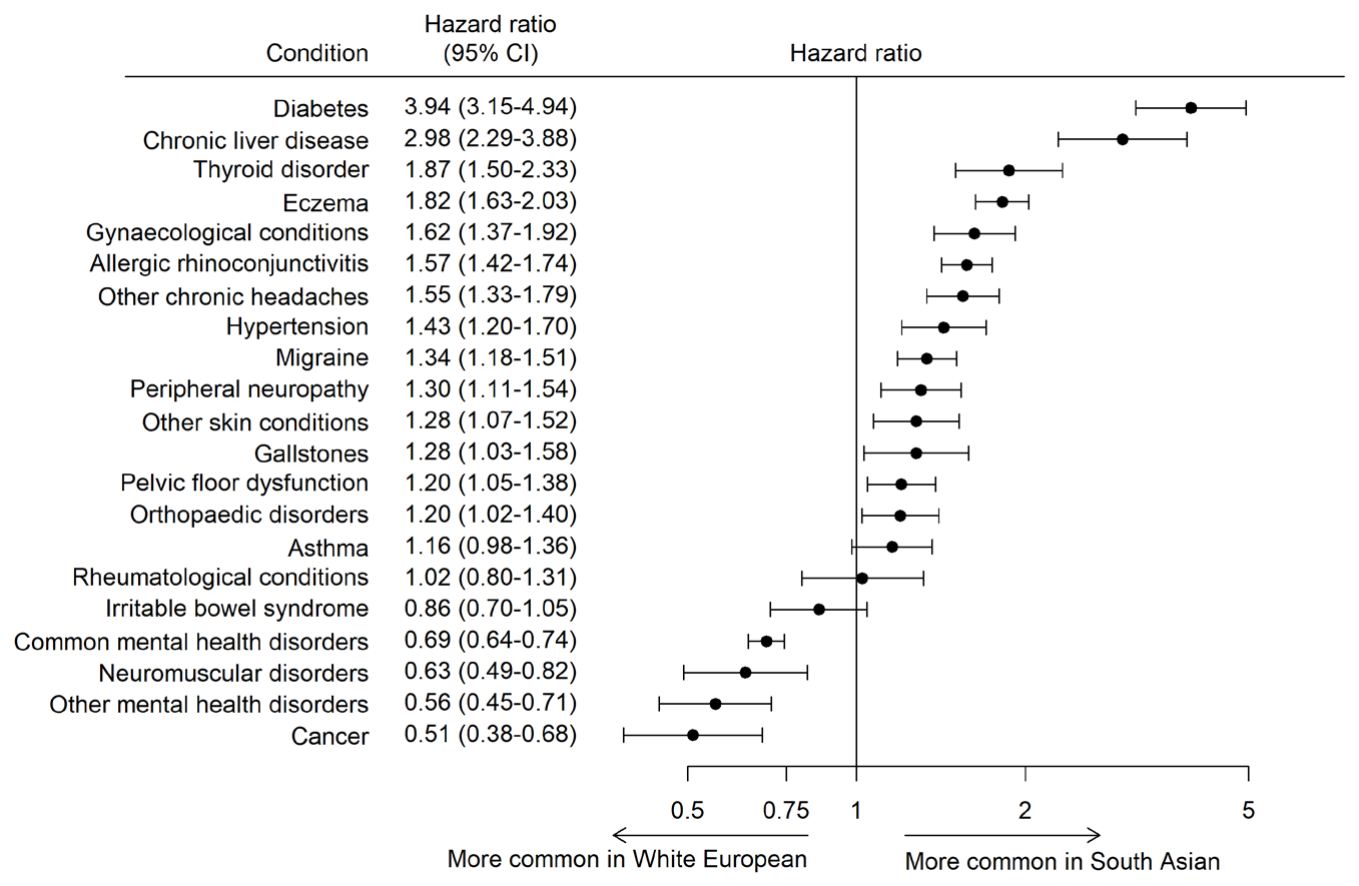
Age-adjusted hazard ratios with 95% CI for common health conditions in South Asian and White European women (White European is the reference).

The HRs for each health condition are shown in Figure 3. Cancer (HR 0.53 [95%CI 0.40, 0.70]), other mental health disorders (HR 0.56 [95%CI 0.44, 0.70]), neuromuscular disorders (HR 0.63 [95%CI 0.49, 0.82]) and common mental health disorders (HR 0.69 [95%CI 0.64, 0.74]) were more common in White European mothers. South Asian mothers were at statistically significantly higher risk of developing 14 out of the 21 health conditions, with diabetes (HR 3.92 [95%CI 3.13, 4.91]) and chronic liver diseases (HR 2.98 [95%CI 2.29, 3.88]) have the largest hazard ratios.

## Discussion

### Main findings

This study describes the prevalence and incidence of 21 common health conditions in mothers participating in the Born in Bradford cohort before and after recruitment and explores differences in the risk of conditions in women of White European and South Asian heritage. Mental health and atopic disorders were the most common health conditions in both ethnic groups. The risk of developing 14 of the 21 health condition was statistically significantly higher in South Asian mothers, with diabetes and chronic liver disease having the greatest difference, at four and three times higher compared to White European women. White European mothers were at higher risk of developing common and other mental health disorders, neuromuscular disorders, and cancer.

### Ethnic inequality

***Mental health disorders*.** The risk of common and other mental health disorders in South Asian women was 31% and 44% lower respectively than in White European women. Previous research on women participating in BiB found that although minority ethnic women self-report similar levels of psychological distress as White British women, they are less likely to access treatment and therefore have a GP-recorded diagnosis ^9^. It is therefore likely that the incidence of common mental health disorders in South Asian women is underestimated using health service data. An alternative explanation may be that stronger social networks evidence in South Asian women may protect against mental ill-health by provision of financial, social or emotional support ^10^.

***Diabetes mellitus*.** We found that women of South Asian heritage were almost four times more likely to develop diabetes compared to White European women. This is comparable to other recent estimates reported in people aged between 40–69 ^11^, though the mean age of women by the end of our study was 40 years, suggesting that differential risk starts young and may yet be higher.

***Chronic liver diseases*.** The development of chronic liver disease in South Asian-heritage women was three times higher than in White European participants, predominantly due to fatty liver disease. A recent systematic review and meta-analysis found that South Asian men and women have more fat in the liver compared to their White European counterparts despite having similar BMI levels ^12^. Although our primary care data did not categorically distinguish between the types of fatty liver disease, the high prevalence of non-alcoholic fatty liver disease (NAFLD) in South Asian-heritage people has been well documented and is associated with obesity, dyslipidaemia, diabetes, and hypertension ^13^, the latter two of which are also reported as being higher in South Asian women in our study.

***Thyroid disorders*.** Women of South Asian heritage were at almost twice the risk of developing thyroid disorders compared to White European women, with hypothyroidism being the most common. Hypothyroidism can be caused by low iodine levels, and a study of almost 7,000 BiB mothers found that the urinary iodine-to-creatinine ratio was lower in Pakistani compared to White European participants ^14^. Another small multi-ethnic study of thyroid function in pregnancy found that women of South Asian heritage had higher levels of serum thyroid stimulating hormone (TSH) compared with ethnic Europeans despite having similar levels of iodine deficiency; a higher prevalence of subclinical hypothyroidism was also observed ^15^.

***Gynaecological disorders*.** We also found that women of South Asian heritage had higher rates of uterine fibroids, infertility and polycystic ovarian syndrome compared to White European women but lower rates of endometriosis. Systematic reviews have reported that black ethnicity was a strong risk factor for uterine fibroids ^16^ although black women are less likely to be diagnosed with endometriosis ^17^. There is limited literature on the prevalence of these disorders in South Asian women apart from a small US-based study that found South Asian women had a similar prevalence of uterine fibroids as white women ^18^.

### Reasons for ethnic inequality and clinical implications

The observed differences in the incidence of most health conditions between ethnic groups can likely be attributed to a combination of social, cultural, genetic, and lifestyle factors ^19– 21^. There is a clear link between socioeconomic deprivation and poor health, and data from England, including our own cohort, indicate that ethnic minorities experience higher deprivation compared to the White population ^19, 21^. Differences in lifestyle behaviours may also contribute to these disparities. While people of South Asian heritage are often less likely to engage in risky behaviours such as smoking and excessive alcohol consumption, studies indicate that they are more likely to be physically inactive, have a poor diet, and experience higher rates of obesity ^22^. Genetic and epigenetic factors may also underlie the risk of some health conditions, such as diabetes (including worse diabetes-related outcomes in South Asian compared to White European people), cardiovascular health conditions, and insulin resistance ^23^. There is also evidence that people of minority ethnic groups have a higher risk of allergic conditions and autoimmune disorders ^24^, with the onset of these condition occurring earlier in non-White compared to White groups ^25^.

Health inequalities are also evident with regards to health access, with ethnic minority patients reporting poorer experience of healthcare services compared to White British groups ^22^. The uptake of population health screening is also lower in ethnic minorities and those living in deprived areas ^26^: screening rates for breast, cervical and bowel cancer are lower in South Asian. This lower uptake likely extends to participation in other health improvement programmes, such as those for diabetes management ^22^. Strategies focusing on improving health literacy and ensuring cultural sensitivity and appropriateness in the healthcare setting have been recommended in an attempt to address these barriers ^22, 26, 27^.

### Strengths and limitations

Using electronic health records, this study provides a comprehensive assessment of the prevalence, incidence, and ethnic differences in risk for 21 non-communicable health conditions in a bi-ethnic sample of 9,784 women enrolled in a longitudinal cohort study. A key strength is the large proportion of South Asian women (52%), which allowed us to identify health inequalities in some conditions that have not previously been widely explored or reported. While to the proportion of South Asian heritage women in the BiB cohort (50%) is higher than their estimated proportion in the general Bradford population (around 22% at the time of enrolment to the BiB study), this reflects the obstetric population attending Bradford Royal Infirmary during the recruitment period, suggesting it is representative of the pregnant women in the area at the time.

To accurately determine prevalent conditions, we implemented a minimum requirement of two years of medical records prior to recruitment. This criterion was based on recorded GP events, which might not perfectly align with continuous registration with GP practice and could have introduced some bias in our prevalence estimates. While exploring the impact of area deprivation on incidence would have been valuable, the numbers if event within specific deprivation strata was insufficient for meaningful analysis. Furthermore, while it would have been preferable to present multivariable hazard ratios adjusted for known lifestyle factors such as physical activity and diet, we unfortunately lacked sufficient information on these potential confounders. We acknowledge that these unmeasured lifestyle factors could influence disease risk and incidence rates, potentially leading to an over- or under-estimation of the impact of ethnicity on health disparities. However, it is important to note that this study is primarily descriptive, aiming to highlight the differences in health conditions between these two groups in the BiB cohort and inform future research directions, rather than to investigate the underlying causes of these differences. We were also limited to studying broader ethnic groups of White European and South Asian due to the small numbers within finer ethnic categories, which may have obscured important intra-group differences. Finally, as this study exclusively examines postpartum women in Bradford, it’s generalisability to other UK populations and to men is limited. However, they are likely to represent parous women in similar urban populations with high levels of deprivation and diversity.

## Conclusion

In conclusion, our findings reveal the early emergence and distinct ethnic patterns of non-communicable health conditions in women from White European and South Asian heritage. The impacts of these health conditions on health services may be greatest in later adulthood, but the opportunity for prevention is greatest in early life. Tackling upstream modifiable risk factors at an individual level (diet, physical activity, smoking) and at a systems level (environment, housing, education, urban design) remains a crucial but neglected priority for improving long term health outcomes and reducing health service pressures ^28^.

## Data Availability

The underlying data for this study is not openly available due to confidentiality issues. However, researchers are encouraged to make use of the BiB data, which are available through a system of managed open access. Before you contact us, please make sure you have read our Guidance for Collaborators ( https://borninbradford.nhs.uk/research/guidance-for-collaborators/). Our BiB Executive reviews proposals on a monthly basis and we endeavour to respond to your request as soon as possible. You can find out about the different datasets in our data dictionary ( https://borninbradford.github.io/datadict/). If you are unsure if we have the data that you need, please contact a member of the BiB team ( borninbradford@bthft.nhs.uk). Please see https://borninbradford.nhs.uk/research/how-to-access-data/ for further information on how to access data.

## Software availability

The analyses in this study was conducted using proprietary software. The equivalent functions are available in R ( https://www.r-project.org/), which is free software for statistical computing and graphics.

## References

[1] British Medical Association: Valuing health. Why prioritising population health is essential to prosperity.2022. Reference Source

[2] Coates MJP Bhardwaj SA Igwe R : The relationship between ethnicity and socioeconomic deprivation as determinants of health: a systematic review. *Epidemiol Public Health.* 2024;2(1): 1030. 10.52768/EpidemiolPublicHealth/1030

[3] MBRRACE-UK: Saving lives, improving mothers care.2024. Reference Source 10.1016/j.ijoa.2015.03.004 25841640

[4] Public Health England: Fingertips.Fingertips.phe.org.uk. Reference Source

[5] City of Bradford Metropolitan District Council: 2021 census: ethnic group, religion and language spoken. 2022. Reference Source

[6] Wright J Small N Raynor P : Cohort profile: the Born in Bradford multi-ethnic family cohort study. *Int J Epidemiol.* 2013;42(4):978–91. 10.1093/ije/dys112 23064411

[7] Lee SI Azcoaga-Lorenzo A Agrawal U : Epidemiology of pre-existing multimorbidity in pregnant women in the UK in 2018: a population-based cross-sectional study. *BMC Pregnancy Childbirth.* 2022;22(1):120. 10.1186/s12884-022-04442-3 35148719 PMC8840793

[8] Office for National Statistics: Ethnic group statistics: a guide for the collection and classification of ethnicity data.London: The Stationary Office,2003, 2003. Reference Source

[9] Prady SL Pickett KE Petherick ES : Evaluation of ethnic disparities in detection of depression and anxiety in primary care during the maternal period: combined analysis of routine and cohort data. *Br J Psychiatry.* 2016;208(5):453–61. 10.1192/bjp.bp.114.158832 26795424 PMC4853643

[10] Uphoff EP Pickett KE Wright J : Social gradients in health for Pakistani and White British women and infants in two UK birth cohorts. *Ethn Health.* 2016;21(5):452–67. 10.1080/13557858.2015.1091442 26428034

[11] Farmaki AE Garfield V Eastwood SV : Type 2 diabetes risks and determinants in second-generation migrants and mixed ethnicity people of South Asian and African Caribbean descent in the UK. *Diabetologia.* 2022;65(1):113–127. 10.1007/s00125-021-05580-7 34668055 PMC8660755

[12] Iliodromiti S McLaren J Ghouri N : Liver, visceral and subcutaneous fat in men and women of South Asian and white European descent: a systematic review and meta-analysis of new and published data. *Diabetologia.* 2023;66(1):44–56. 10.1007/s00125-022-05803-5 36224274 PMC9729139

[13] Niriella MA Ediriweera DS Withanage MY : Prevalence and associated factors for non-alcoholic fatty liver disease among adults in the South Asian Region: a meta-analysis. *Lancet Reg Health Southeast Asia.* 2023;15: 100220. 10.1016/j.lansea.2023.100220 37614359 PMC10442973

[14] Threapleton DE Snart CJP Keeble C : Maternal iodine status in a multi-ethnic UK birth cohort: associations with child cognitive and educational development. *Paediatr Perinat Epidemiol.* 2021;35(2):236–246. 10.1111/ppe.12719 32870514

[15] Sletner L Jenum AK Qvigstad E : Thyroid function during pregnancy in a multiethnic population in Norway. *J Endocr Soc.* 2021;5(7): bvab078. 10.1210/jendso/bvab078 34159284 PMC8212686

[16] Stewart EA Cookson CL Gandolfo RA : Epidemiology of uterine fibroids: a systematic review. *BJOG.* 2017;124(10):1501–1512. 10.1111/1471-0528.14640 28296146

[17] Bougie O Yap MI Sikora L : Influence of race/ethnicity on prevalence and presentation of endometriosis: a systematic review and meta-analysis. *BJOG.* 2019;126(9):1104–1115. 10.1111/1471-0528.15692 30908874

[18] Marsh EE Ekpo GE Cardozo ER : Racial differences in fibroid prevalence and ultrasound findings in asymptomatic young women (18–30 years old): a pilot study. *Fertil Steril.* 2013;99(7):1951–7. 10.1016/j.fertnstert.2013.02.017 23498888 PMC4465811

[19] Public Health England: Local action on health inequalities: understanding and reducing ethnic inequalities in health. 2018. Reference Source

[20] Bradby H Nazroo JY : Health, ethnicity, and race.In: *The Wiley Blackwell Companion to Medical Sociology.* 2021. 10.1002/9781119633808.ch13

[21] Kapdia D Zhang J Salway S : Ethnic inequalities in healthcare: a rapid evidence review. 2022. Reference Source

[22] Raleigh V Holmes J : The health of people from ethnic minority groups in England. 2021. Reference Source

[23] Hanif W Susarla R : Diabetes and cardiovascular risk in UK South Asians: an overview. *Br J Cardiol.* 2018;25(suppl 2):S8–S13. 10.5837/bjc.2018.s08

[24] Subramanian A Adderley NJ Gkoutos GV : Ethnicity-based differences in the incident risk of allergic diseases and autoimmune disorders: a UK-based retrospective cohort study of 4.4 million participants. *Clin Exp Allergy.* 2021;51(1):144–147. 10.1111/cea.13741 32946613

[25] Sharma-Oates A Zemedikun DT Kumar K : Early onset of immune-mediated diseases in minority ethnic groups in the UK. *BMC Med.* 2022;20(1): 346. 10.1186/s12916-022-02544-5 36224602 PMC9558944

[26] Public Health England: PHE screening inequalities strategy. 2020. Reference Source

[27] Patel KCR Hanif W : Ethnic health inequalities in the NHS. *BMJ.* 2022;376:o607. 10.1136/bmj.o607

[28] Wright J Hayward A West J : ActEarly: a city collaboratory approach to early promotion of good health and wellbeing [version 1; peer review: 2 approved]. *Wellcome Open Res.* 2019;4: 156. 10.12688/wellcomeopenres.15443.1 31840089 PMC6904987

