# Supplementary material for: The emergence of common health conditions across the life course: evidence from the Born in Bradford family cohort
Source: Wellcome Open Res. 2025 May 16;9:210. Originally published 2024 Apr 18. [Version 2] doi: 10.12688/wellcomeopenres.20992.2 (PMC12134723; doi:10.12688/wellcomeopenres.20992.2)
Supplement: Peer review history

# Reviewer report: Reviewer response for version 2

**DOI:** 10.21956/wellcomeopenres.26688.r123716

**Published:** 2025-06-03

**Authors:** Syed Masud Ahmed

**Affiliations:** 1 BRAC University, Dhaka, Dhaka Division, Bangladesh

Thanks to the authors for responding to the comments/suggestions and making necessary changes.

 I have no further comments. Tha manuscript can be accepted for indexing.

 Thanks.

Is the work clearly and accurately presented and does it cite the current literature?

Yes

If applicable, is the statistical analysis and its interpretation appropriate?

I cannot comment. A qualified statistician is required.

Are all the source data underlying the results available to ensure full reproducibility?

No

Is the study design appropriate and is the work technically sound?

Yes

Are the conclusions drawn adequately supported by the results?

Partly

Are sufficient details of methods and analysis provided to allow replication by others?

Partly

Reviewer Expertise:

NA

I confirm that I have read this submission and believe that I have an appropriate level of expertise to confirm that it is of an acceptable scientific standard.

# Reviewer report: Reviewer response for version 2

**DOI:** 10.21956/wellcomeopenres.26688.r123717

**Published:** 2025-06-02

**Authors:** Nusrat Husain

**Affiliations:** 1 Division of Psychology & Mental Health, The University of Manchester, Manchester, UK

The author has addressed all the points raised in my review report. I believe these revisions have enhanced the overall quality of the manuscript, making it more informative and valuable for readers.

Is the work clearly and accurately presented and does it cite the current literature?

Yes

If applicable, is the statistical analysis and its interpretation appropriate?

Partly

Are all the source data underlying the results available to ensure full reproducibility?

Yes

Is the study design appropriate and is the work technically sound?

Yes

Are the conclusions drawn adequately supported by the results?

Partly

Are sufficient details of methods and analysis provided to allow replication by others?

Yes

Reviewer Expertise:

Global Mental Health and Cultural Psychiatry

I confirm that I have read this submission and believe that I have an appropriate level of expertise to confirm that it is of an acceptable scientific standard.

# Reviewer report: Reviewer response for version 1

**DOI:** 10.21956/wellcomeopenres.23225.r107111

**Published:** 2024-11-13

**Authors:** Nusrat Husain

**Affiliations:** 1 Division of Psychology & Mental Health, The University of Manchester, Manchester, UK

The Born in Bradford (BiB) study leverages a large, diverse family cohort with linked routine health records to examine health disparities between South Asian and White European populations in the UK. By focusing on 21 common health conditions, the study reports the incidence and prevalence of these diseases and evaluates differences across ethnic groups, thereby contributing to our understanding of health inequalities.

 This is a large, Ethnically Diverse Sample with nearly 10,000 participants, thus, allowing for meaningful comparisons between South Asian and White European women. The cohort study longitudinal design, spanning over a decade, enhances reliability in estimating disease incidence over time and enables tracking of health trajectories. Use of Routine Health Records (EHR) allows for comprehensive data capture on disease diagnosis and medication history, reducing potential recall bias associated with self-reported health conditions.

 The study has focused on ethnic health disparities, highlighting areas where public health interventions can be tailored to address ethnic-specific health needs.

 However, while the findings are insightful, there are some areas where the study's methodology and interpretation need a bit more explanation.

  

- The ethnic minorities are considered a hard-to-engage group. The study does not discuss potential biases in recruitment or participation rates between ethnic groups. If participation differs significantly due to socioeconomic factors, cultural differences, or healthcare access, it could skew the results. I notice that the study reports age-adjusted hazard ratios (HRs) but does not adjust for other important variables like socioeconomic status, lifestyle (e.g., physical activity, diet), or healthcare access in any detail. These confounders could affect disease risk and incidence rates, potentially leading to an over- or underestimation of ethnicity's impact on health disparities.
- Hazard ratios (HR) are presented without much discussion on the clinical significance and resource implications. For instance, while the HR for diabetes (3.94) among South Asian women is markedly high, the clinical implications or potential explanations for such a strong association are not explored in detail.

- The study categorizes participants into broad ethnic groups (South Asian vs. White European), potentially obscuring important intra-group differences. South Asians, for instance, include diverse populations (e.g., Pakistani, Indian, Bangladeshi) with varied genetic, cultural, and socioeconomic backgrounds, which could influence disease risk and healthcare behaviour. For example, previous reports such as Weich et al. 2004, and Gater et al. 2009, suggested significantly higher rates of depression compared to the majority white population. In my opinion including the Irish population could also have an impact on results.

- Although the study mentions cultural and lifestyle factors as potential explanations, it does not empirically investigate these aspects. The study focuses on 21 conditions but does not justify this selection. Certain conditions with known ethnic disparities, such as cardiovascular diseases (other than diabetes), are not included, which could have strengthened the study.

- I suggest as the study exclusively examines women in Bradford, limiting its generalizability to other UK populations or to men should be discussed in a bit more detail.

I suggest the authors discuss the implications of these findings in context with addressing ethnic inequalities which have been reported for many years but there has been little work on developing interventions. In my opinion, this is an excellent study and a wider discussion with earlier studies findings may help in working towards developing appropriate interventions.

Is the work clearly and accurately presented and does it cite the current literature?

Yes

If applicable, is the statistical analysis and its interpretation appropriate?

Partly

Are all the source data underlying the results available to ensure full reproducibility?

Yes

Is the study design appropriate and is the work technically sound?

Yes

Are the conclusions drawn adequately supported by the results?

Partly

Are sufficient details of methods and analysis provided to allow replication by others?

Yes

Reviewer Expertise:

Global Mental Health and Cultural Psychiatry

I confirm that I have read this submission and believe that I have an appropriate level of expertise to confirm that it is of an acceptable scientific standard, however I have significant reservations, as outlined above.

# Response

**Published:** 2024-12-06

**Authors:** Gillian Santorelli

**Affiliations:** Bradford Hospitals National Health Service Trust, UK

We thank the reviewer for taking the time to read our paper. In response to your comments:

 1.  Between 2007 – 2011, at the time of recruitment to BiB, 50% of the mothers recruited to BiB were of South Asian ethnicity compared to 47% of non-cohort births, reflecting our success in recruiting women from ethnic minorities. As described in the response to reviewer 1, this was a descriptive rather than explanatory study. This, together with the lack of comprehensive lifestyle data (e.g. physical activity, diet) explains why we presented HR adjusted only for age. We will clarify this in the manuscript.

 2. We have discussed ethnic disparities in those health conditions with substantial differences between groups in the *Discussion*. As previously mentioned, the goal of this study was descriptive, aimed at identifying patterns in disease incidence across ethnic groups. This serves as a foundation for further exploration of diseases with notable differences between groups.

 3. The majority of the South Asian group (85%) were of Pakistani heritage, leaving the numbers of women of Indian and Bangladeshi heritage too small to yield meaningful results. Of the White European population, 95% were White British. We believe that the small representation of other ethnicities within each group is unlikely to have influenced the results significantly.

 4. The rationale for selecting the diseases discussed in this paper detailed under *Outcomes* in the *Methods* section: “Following exploratory analysis of the incidence of various health conditions, we report trends for 21 health conditions (19 physical and 2 mental health) with an incidence per 1000 person-years ≥2% in either ethnic group.” While 79 health conditions were initially identified as part of another study (MuM-PrediCT), this has not been explained in the current manuscript. We will revise the text to include this information.

 5. We have acknowledged that the findings are Bradford-specific to parous women only

# Reviewer report: Reviewer response for version 1

**DOI:** 10.21956/wellcomeopenres.23225.r107115

**Published:** 2024-11-04

**Authors:** Syed Masud Ahmed

**Affiliations:** 1 BRAC University, Dhaka, Dhaka Division, Bangladesh

This is a well-designed study using a cohort of reproductive age women to measure the prevalence and incidence of chronic diseases over time. A timely study to inform the design of interventions to modify possible risk factors to prevent chronic diseases in future. However, there are specific issues which need attention:

 1. **Introduction: **The current text in the intro, if fact, is part of the methods section, describing the data cohort; a brief literature review text should identify the problems to be addressed, including gaps focused in the study, and the objectives; Justification/rationale of the study; “value addition” to answer the question: so what? 

 2. **Methods:** Beside the description of the BiB dataset, the methods should describe in greater detail the process of recruitment, categories of health conditions studied and underlying reasons for selecting these diseases, the details of the analysis process, etc. Also, a brief description of sociodemographic and life-style characteristics of the two ethnic groups that may influence disease outcomes is needed. The statistical procedures use should be checked by a senior statistician to confirm validity of analysis (including elaborating the statistical method used e.g., the HR how it is composed and what’s its significance etc.

 3. **Interpretation and Discussion: **This section could be enhanced by comparing the SA pop characteristics with those availing in their origin country and entangling how migration changed the characteristics giving rise to ne epidemiological transition etc.

Is the work clearly and accurately presented and does it cite the current literature?

Yes

If applicable, is the statistical analysis and its interpretation appropriate?

I cannot comment. A qualified statistician is required.

Are all the source data underlying the results available to ensure full reproducibility?

No

Is the study design appropriate and is the work technically sound?

Yes

Are the conclusions drawn adequately supported by the results?

Partly

Are sufficient details of methods and analysis provided to allow replication by others?

Partly

Reviewer Expertise:

NA

I confirm that I have read this submission and believe that I have an appropriate level of expertise to confirm that it is of an acceptable scientific standard, however I have significant reservations, as outlined above.

# Response

**Published:** 2024-12-06

**Authors:** Gillian Santorelli

**Affiliations:** Bradford Hospitals National Health Service Trust, UK

We thank the reviewer for taking time to read our paper. In response to your comments:

 1. We will expand the introduction to include a literature review and clarify our objectives, as suggested. The aim of this study was descriptive, focusing on identifying patterns in disease incidence across ethnic groups. This provides a baseline for further investigation into diseases that show significant differences between two groups. Our study is distinctive in its comprehensive examination of a wide range of health conditions, which has not been previously explored in the same bi-ethnic population.

 2.  We have provided a reference outlining the recruitment process, but we will elaborate on this in the introduction. The rationale for selecting the diseases discussed in this paper detailed under *Outcomes* in the *Methods* section: “Following exploratory analysis of the incidence of various health conditions, we report trends for 21 health conditions (19 physical and 2 mental health) with an incidence per 1000 person-years ≥2% in either ethnic group.” While 79 health conditions were initially identified as part of another study (MuM-PrediCT), this has not been explained in the current manuscript. We will revise the text to include this information. Table 2 presents key participant characteristics (age, BMI, smoking status, IMD). Although we would have liked to incorporate other lifestyle factors, such as diet and physical activity, there was significant missing data for these variables. We will describe the purposes and function of the hazard ratio in the statical analysis section.

 3. Thank you for your suggestion of comparing the SA population characteristics to those availing in their origin country, we will add this to our discussion.
